# *Dermacentor reticulatus*: a vector on the rise

**DOI:** 10.1186/s13071-016-1599-x

**Published:** 2016-06-01

**Authors:** Gábor Földvári, Pavel Široký, Sándor Szekeres, Gábor Majoros, Hein Sprong

**Affiliations:** Department of Parasitology and Zoology, Faculty of Veterinary Science, Szent István University, Budapest, Hungary; Department of Biology and Wildlife Diseases, Faculty of Veterinary Hygiene and Ecology, University of Veterinary and Pharmaceutical Sciences Brno, Brno, Czech Republic; CEITEC-Central European Institute of Technology, University of Veterinary and Pharmaceutical Sciences Brno, Brno, Czech Republic; National Institute of Public Health and the Environment, Bilthoven, The Netherlands

**Keywords:** *Dermacentor reticulatus*, Ecology, Geographical distribution, Spread, Epidemiology, Host associations, Europe, Asia, *Babesia canis*, Omsk haemorrhagic fever virus

## Abstract

*Dermacentor reticulatus* is a hard tick species with extraordinary biological features. It has a high reproduction rate, a rapid developmental cycle, and is also able to overcome years of unfavourable conditions. *Dermacentor reticulatus* can survive under water for several months and is cold-hardy even compared to other tick species. It has a wide host range: over 60 different wild and domesticated hosts are known for the three active developmental stages. Its high adaptiveness gives an edge to this tick species as shown by new data on the emergence and establishment of *D. reticulatus* populations throughout Europe. The tick has been the research focus of a growing number of scientists, physicians and veterinarians. Within the Web of Science database, more than a fifth of the over 700 items published on this species between 1897 and 2015 appeared in the last three years (2013–2015). Here we attempt to synthesize current knowledge on the systematics, ecology, geographical distribution and recent spread of the species and to highlight the great spectrum of possible veterinary and public health threats it poses. Canine babesiosis caused by *Babesia canis* is a severe leading canine vector-borne disease in many endemic areas. Although less frequently than *Ixodes ricinus*, *D. reticulatus* adults bite humans and transmit several *Rickettsia* spp., Omsk haemorrhagic fever virus or Tick-borne encephalitis virus. We have not solely collected and reviewed the latest and fundamental scientific papers available in primary databases but also widened our scope to books, theses, conference papers and specialists colleagues’ experience where needed. Besides the dominant literature available in English, we also tried to access scientific literature in German, Russian and eastern European languages as well. We hope to inspire future research projects that are necessary to understand the basic life-cycle and ecology of this vector in order to understand and prevent disease threats. We conclude that although great strides have been made in our knowledge of the eco-epidemiology of this species, several gaps still need to be filled with basic research, targeting possible reservoir and vector roles and the key factors resulting in the observed geographical spread of *D. reticulatus*.

**Electronic supplementary material:**

The online version of this article (doi:10.1186/s13071-016-1599-x) contains supplementary material, which is available to authorized users.

## Background

An ideal arthropod vector has a high reproduction rate, an ability to survive and even spread within variable habitats and an opportunity to host and transmit a great variety of pathogens. All these conditions are perfectly met by the hard tick species *Dermacentor reticulatus*. Fertilised females lay up to 7,200 eggs [1] and adults possess an extreme tolerance to the changing environment. Adapted to river basins among other habitats, they survive under water containing organic residues for up to one month and in cool and clean water for more than 100 days [2]. In contrast to dipteran vectors, adult *D. reticulatus* specimens have a long lifespan; they have been shown to survive for up to four years without taking a blood meal [3]. They are even able to tolerate -10 °C for 150 days under laboratory conditions [4] and are shown to be active during the winter in many climatic zones at temperatures at which *Ixodes ricinus* is not active [5]. Furthermore, the speed of its developmental rate from larvae to nymphs and again to adults surpasses *I. ricinus* [6]. *Dermacentor reticulatus* attaches and feeds on a wide range of hosts, including wild and domesticated mammals, for days at a time enabling the tick to spread over large distances. The high adaptiveness of this species is exemplified by the recent new establishments of *D. reticulatus* populations in many countries and regions of Europe. The multitude of pathogens that can (potentially) be transmitted by this vector highlights the long-shared evolutionary history of several viruses, bacteria and protists with *D. reticulatus* and its hosts. First of all, it is a vector of pathogens causing animal health problems. Canine babesiosis caused by *Babesia canis* is a severe leading canine vector-borne disease in many endemic areas. Although less frequently than *I. ricinus*, *D. reticulatus* adults bite humans and can transmit several *Rickettsia* spp., Omsk haemorrhagic fever virus and tick-borne encephalitis virus. Here we attempt to summarize current knowledge on the systematics, ecology, geographical distribution and recent expansion of *D. reticulatus*, and highlight the great spectrum of possible veterinary and public health threats posed by this tick species, which is currently invading new areas.

### Systematics

*Dermacentor reticulatus* (Fabricius, 1794) is a metastriate tick species belonging to the almost globally cosmopolitan genus *Dermacentor* (consisting of 35 currently recognised species), subfamily Rhipicephalinae, family Ixodidae, order Ixodida, subclass Acari, class Arachnida [7, 8]. It has previously been known by several junior synonym names (see e.g. Guglielmone and Nava [9]), with *Dermacentor pictus* (Hermann, 1804) as one of the most widespread of these, especially in the former Soviet Union and eastern Europe [10]. It was originally named *Acarus reticulatus* Fabricius, 1794 and given its current status by Koch in 1844 [11]. English names of this species used in scientific publications are ornate cow tick [12], ornate dog tick [13], meadow tick [14] or marsh tick [15–17].

*Dermacentor reticulatus* can be unambiguously distinguished from *D. marginatus*, despite its morphological resemblance [18]. Although a bit smaller than *D. marginatus*, *D. reticulatus* is considerably larger than most *Ixodes* and *Haemaphysalis* ticks. Males (4.2–4.8 mm) are larger than females (3.8–4.2 mm) when unfed, however a fully fed female reaches 1 cm [12]. Nymphs are 1.4–1.8 mm and larvae only 0.5 mm in size [4].

**Fig. 1:**
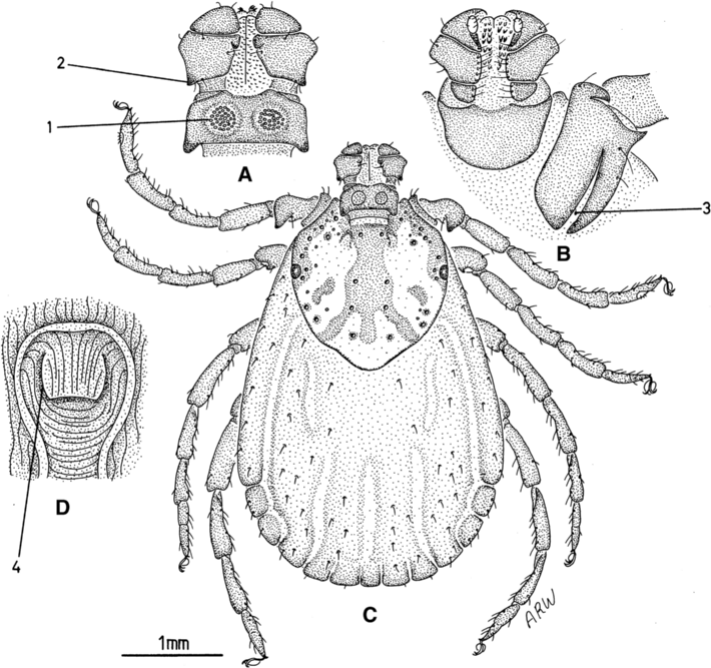
Most important morphological characters of female *Dermacentor reticulatus*. **a** Dorsal capitulum. **b** Ventral coxa. **c** Dorsal body. **d** Genital aperture. 1, Porose areas shape is a broad oval (nearly circular). 2, Palp articles 2 posterior spur is present on the dorsal surface. 3, Coxae 1 gap between external and internal spurs is narrow (also the external spur is as long as the internal spur). 4, Genital aperture posterior lips have a broad U shape (this shape is truncated posteriorly). Original drawings by Alan R. Walker [13]

**Fig. 2:**
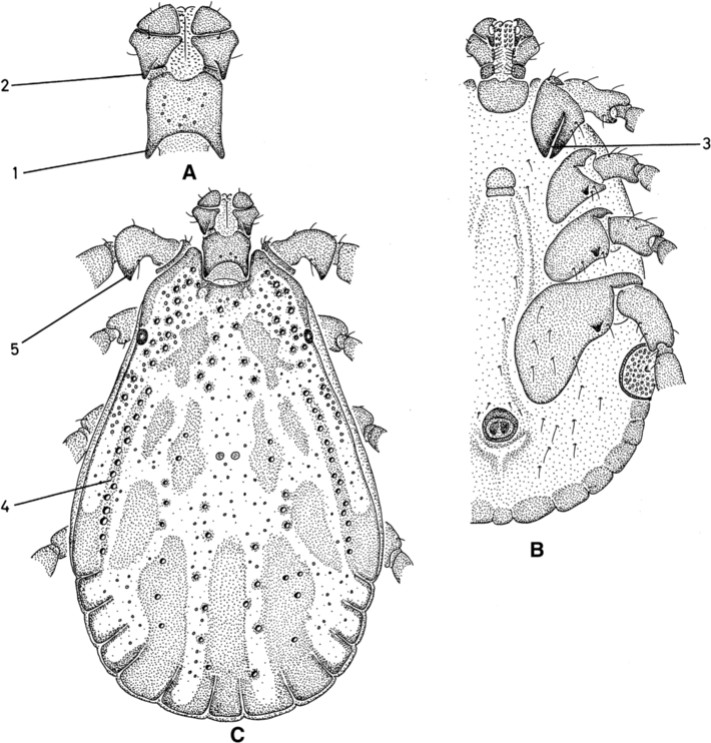
Most important morphological characters of male *Dermacentor reticulatus.* **a** Dorsal capitulum. **b** Ventral body. **c** Dorsal body. 1, Cornua length is long. 2, Palp articles 2 posterior spur is long on the dorsal surface. 3, Coxae 1 gap between external and internal spurs is narrow (also the external spur is as long as the internal spur). 4, Lateral groove type is in the form of punctations only (there is no groove visible). 5, Trochanter 1 posterior spur is long on the dorsal surface. Original drawings by Alan R. Walker [13]

**Fig. 3:**
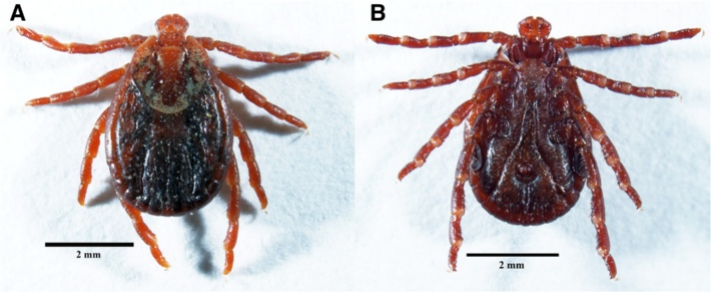
Photomicrograph of female *Dermacentor reticulatus*. **a** Dorsal view. **b** Ventral view

**Fig. 4:**
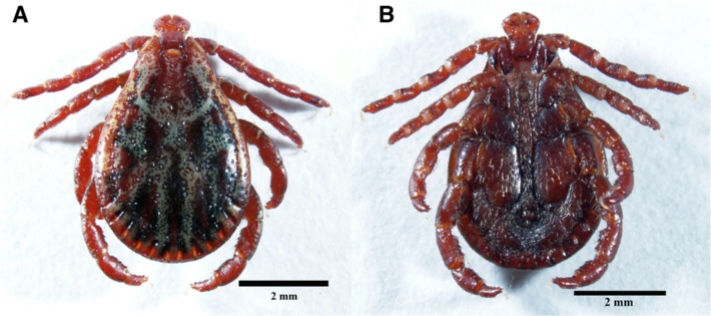
Photomicrograph of male *Dermacentor reticulatus*. **a** Dorsal view. **b** Ventral view

**Fig. 5:**
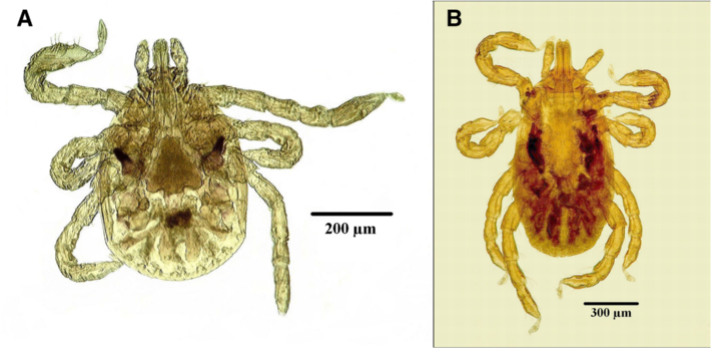
Photomicrograph of *Dermacentor reticulatus*. **a** Larva. **b** Nymph

There are excellent keys [12, 13] available for the morphological identification of adults using some key features such as the palps and coxae, as shown on Figs. 1, 2, 3 and 4. Like all species of *Dermacentor*, *D. reticulatus* has relatively short mouthparts with a basis capitulum of straight lateral margins, both sexes have white enamel ornamentation and the males have very large fourth coxae [10, 12, 13]. As *D. reticulatus* can sometimes be found on the same host with *D. marginatus*, differentiation of the two species is important. For both sexes, the most important feature is the presence of a palpal spur in *D. reticulatus* (*vs* absent in *D. marginatus*) (Figs. 1 and 2). For females, most prominent details are the shape of porose areas, the shape of the gap between internal and external spurs on coxa I and the size of the lips in the genital aperture (Fig. 1). For males, cornua are long in *D. reticulatus* (*vs* short in *D. marginatus*) and the lateral groove is in the form of punctations only in *D. reticulatus* (no groove visible, see Fig. 2) [13]. Compared to adults, larvae (Fig. 5a) and nymphs (Fig. 5b), are difficult to identify. They resemble *Rhipicephalus* spp. immatures, especially when engorged. Usually, *D. reticulatus* immatures are only accessible as engorged specimens because they cannot be collected from the vegetation (see also section “Life-cycle and ecology” below). Engorgement modifies their morphological characters and identification requires mounting and careful examination of the specimen under light microscopy by an experienced entomologist (Fig. 5).

### Life-cycle and ecology

Our current understanding of life-cycle traits and ecological aspects of *D. reticulatus* is rather limited compared to the well-studied *Ixodes ricinus* or *Ixodes scapularis*. This may be partly due to the hidden nature of the larvae and nymphs of this species. Although it is a three-host tick, as is *I. ricinus*, in contrast to the latter species the larvae and nymphs of *D. reticulatus* are reportedly nidicolous, while adults show an exophilic (non-nidicolous) behaviour [10, 19, 20]. For this reason, immatures, in contrast to adults, are rarely collected by flagging, with some exceptions [5]. Adults, in contrast, are easy to collect by flagging where they are abundant, and thus it is easier to gain phenology data for them. To assess seasonality or population dynamics of immatures, their preferred hosts have to be investigated.

Larvae usually appear on small mammal hosts in May-June, with their highest abundance in June-July in temperate Europe [6, 10, 19, 20]. *Dermacentor reticulatus* was shown to have a higher developmental rate compared to *I. ricinus* but a relatively low mortality rate [10]. Engorged larvae moult and give rise to feeding nymphs within a month and the whole generation is completed within a few months in nature [6]. The relatively rapid development is also obvious from a table that lists 37 hard tick species maintained in the laboratory, among which *D. reticulatus* was shown to have one of the shortest life-cycles [7]. The fact that nymphs are usually active only for one month (July-August) [6, 19, 20] results in a very small window of opportunity for co-feeding larvae and nymphs. Whereas only 3 % of *I. ricinus* nymphs were recorded on hosts without conspecific larvae, 28 % of *D. reticulatus* nymphs occurred in the absence of larvae [6]. Another difference from the ecology of *I. ricinus* is that while *I. ricinus* nymphs and larvae feeding on the same host probably represent two different generations, separated in age by a year, *D. reticulatus* nymphs and larvae are part of the same generation, maturing within the same summer [21]. Laboratory data show that larvae feed for 2.5–6 days and nymphs for 4–12 days [22–24]. The large accumulation of endosomes (or inclusion bodies in the gut epithelium) [7] during immature feeding and rapid digestion is associated with their short premoulting period [23]. In the more slowly moulting *I. ricinus,* food inclusions are only formed after detachment [23].

**Fig. 6:**
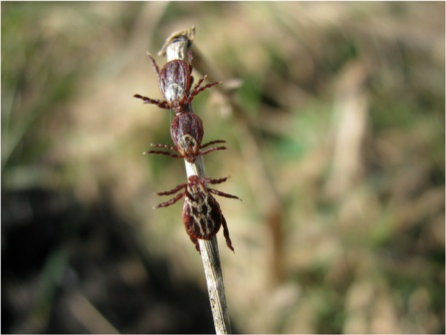
Two female and a male host seeking *Dermacentor reticulatus*

Adults are mainly active from March with a peak in April; they are less abundant during the summer (they completely disappear from vegetation in continental climates) and have a second activity peak in September-October [25–27]. During the winter they undergo diapause which is different from quiescence and is defined as a neurohormonally-mediated dynamic state of low metabolic activity [28]. The relatively early activation of *D. reticulatus* adults after winter diapause is associated with their ability to withstand low temperatures [29] which results in an evolutionary advantage compared to other ticks. Adult *D. reticulatus* follow an ambush strategy to find their hosts [30]. They climb onto weeds, grasses, bushes, or other leafy vegetation (as shown in Additional file 1: Video 1, Additional file 2: Video 2) to wait for passing hosts. The average height for this questing behaviour is 55 cm [19]. Since adult female and male specimens of *D. reticulatus* are three and five times larger respectively than *I. ricinus* [5], they are often visible at the tips of the vegetation (Fig. 6) and can be easily collected by hand during their activity period. Because of their highly sensitive chemical receptors [30] they are attracted by host odours, and are therefore often associated with tracks used by wildlife, dogs and humans [5, 31]. At preferred sites the number of adults collected per hour per flag can reach 222 [32]. Many authors observed female predominance in questing tick populations [5, 33–35] possibly resulting from their metastriate mating strategy, i.e. males need to find a host and tend to spend more time on the host while fertilising several females [7, 36]. Furthermore, females of *D. reticulatus* may have a higher survival rate as they were shown to be more resistant to desiccation than males, as proven in laboratory experiments [37]. In addition, females were shown to predominate also in artificially bred groups of *D. reticulatus* [19] even in lines derived from single, fertilised females suggesting the existence of a genetic mechanism.

Adults prefer medium-sized and large mammals and tend to feed in clusters, resulting in macroscopically visible lesions with local inflammation [38]. The aggregated feeding is probably a consequence of aggregation-attachment pheromones as shown in other metastriate ticks [7] but not studied in *D. reticulatus*. Females feed for 7–15 days under laboratory conditions [1, 22–24, 38, 39]. Balashov [23] noted that *D. reticulatus* females usually attach on the first day but 2–3 days are needed in autumn and winter leading to 1–2 days longer feeding in autumn and 3–4 days longer feeding in winter compared to spring. Overwintering on the host, an unusual trait among three-host ticks, has also been reported for this species [40]. Ticks were observed to remain attached on domestic animals from autumn until the onset of warm spring weather, during which period they do not feed [23]. Another impressive trait is the amount of blood ingested. Although larger species (e.g. *D. marginatus* or *Hyalomma* spp.) are able to take larger blood meals, *D. reticulatus* is the only one for which it has been observed that its faecal weight during feeding may exceed that of the engorged tick [23].

**Fig. 7:**
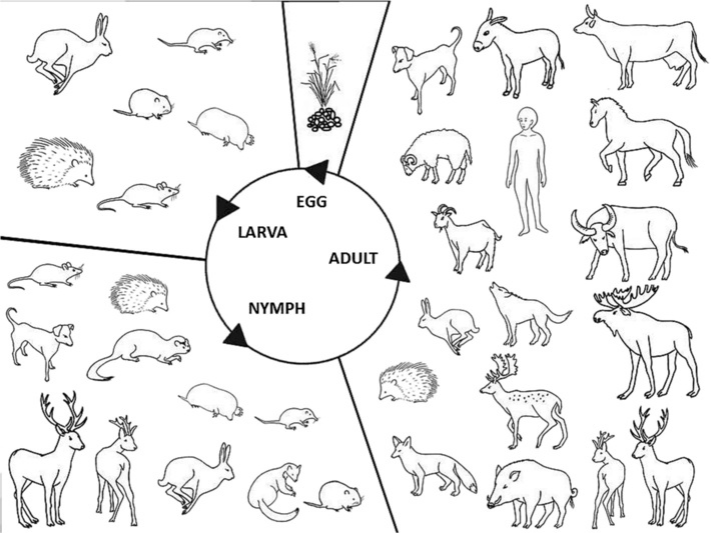
Life-cycle of *Dermacentor reticulatus*

Male individuals also attach and are able to feed for 3–5 days [23] (Hans Dautel, personal communication) and fertilise females exclusively on the host. *Dermacentor reticulatus* males remain on the host for 2–3 months [23] and are considered important vectors of several pathogenic agents due to their intermittent feeding behaviour which is a relevant epidemiological difference compared with *Ixodes* spp. males. Fully-fed fertilised females drop to the ground and lay 3,000–7,200 eggs while covering them with the secretion of the Gené’s organ, protecting the eggs from drying out [1, 41]. Oviposition lasts for 6–25 days and the new generation of larvae will hatch from the egg batch after 12–19 days [22]. The whole life-cycle (Fig. 7) can be completed within the same year or, if the unfed adults overwinter (behavioural diapause), within two years [22]. Nosek [42] observed that usually unfed adults overwinter, however, overwintering of engorged females, engorged nymphs and engorged larvae also occurred during the six-year study [42]. If engorged nymphs overwinter and moult during next spring, the size of the resulting adult is considerably smaller compared to average [42]. Overwintered engorged females are also smaller in size and weight. [42] According to the six years of observation, overwintering unfed females represented the general life-cycle and the overwintering of engorged females, nymphs and larvae was observed less frequently, e.g. spring emergence of freshly moulted adults occurred in 5 % of individuals [42]. Both behavioural and developmental diapause described in this species are obviously biological adaptations to increase chances for survival and consequently to prolong the tick lifespan [23].

All stages of *D. reticulatus* are more seasonal compared to *I. ricinus*. However, if the winter is relatively mild, adults of the former are active throughout the year [5, 25, 43]. During a 24-hour monitoring of questing at a marsh site in March in Wales the minimum temperature at which *D. reticulatus* adults were recorded active was 3.3 °C (at 9 am) and the minimum overnight temperature was -5.4 °C with some adults being active even when the underlying sand surface was frozen. The questing-temperature limit also depends on the tick’s physiological age (Olaf Kahl, personal communication). However, considerable variation can be observed in the seasonal activity of adults according to differences in climatic conditions. It has experimentally been observed that adults were still alive 2.5 years after moulting (third spring) indicating a great tolerance to starvation [44]. According to Olsuf’ev [3], adults can survive for as long as 3–4 years in the absence of hosts in nature [3]. At the eastern end of its range (western Siberia) adults were only active during the brief spring (April-June) with brief autumn activity occurring almost immediately afterwards (July-September) and no activity during the rest of the year. At the western end of the range (Wales, France), they were active for most of the year with a short summer diapause (two months, June-August) and a brief winter period of inactivity (one month, December-January) [5, 45]. The latter does not qualify as a true diapause [46] since *D. reticulatus* adults can reappear relatively quickly during warmer winter days [5, 33]. The winter diapause found in central Europe [22], eastern Europe and western Siberia [33] is likely to be a response to the harsh winter conditions, while such a diapause is not required on the western limit of the species range. In temperate Europe, adults are most active in April-May, activity declines during summer, and there is a second, usually smaller activity peak in September-October [4, 19, 22, 25, 26, 33, 45]. Photoperiod is clearly of underlying importance in controlling behavioural diapause (i.e. suppression of host-seeking activity) [47, 48]. It may be that there is an East-West cline in the inductive photoperiod. Alternatively, the diapause in Europe may result from a temperature-photoperiod interaction in which mild winter conditions are not sufficient to induce diapause [5]. Cessation of adult questing activity at the end of spring may be associated with temperatures but reactivation in the autumn occurs before temperatures fall indicating the importance of photoperiod (decreasing daylight) [5].

During a field study in Wales, UK [5] the observed *D. reticulatus* population exhibited a plastic behavioural response (variation in seasonal activity) within a local area. Macro-temperature appeared to have exerted the predominant influence on ticks at the dune sites, whereas photoperiod was the only macroclimate variable with a significant effect on activity at the marsh site. A microclimatic effect of vegetation temperature and humidity on tick activity was found at the dune site and only vegetation temperature had an effect at the marsh site. Such variation in behaviour within a population is likely to reflect individual responses to microenvironmental cues, i.e. phenotypic plasticity of the species.

Larvae and nymphs usually use the same, predominantly small, mammalian host (Table 1; Fig. 7) for their blood meal. In Europe, *D. reticulatus* immatures are found at higher mean intensity and prevalence on voles than on mice [5, 6, 19–21]. This host-association is the opposite to that in *I. ricinus* which occurs more frequently on mice compared to voles [20, 49]. Hedgehogs, shrews, moles, hares and rabbits are typical hosts, and birds [50, 51] can be occasional hosts for *D. reticulatus* larvae, while nymphs, in addition to these hosts, might feed on weasels, polecats, cervids, goats, dogs [10, 12, 22, 52] and occasionally on birds [22, 50, 51, 53] and humans [4, 54–56]. Szymanski [33] suggested that different species may act as the main host depending on geographical location and habitat type. In open areas in Siberia, the narrow-headed vole (*Microtus gregalis*) was the main host, whereas in forest areas, root voles (*Microtus oeconomus*), northern red-backed voles (*Myodes rutilus*) and common shrews (*Sorex araneus*) were the main hosts. Host species was of more importance than host abundance at study sites in Poland. Although the common shrew was the most abundant host, root voles and field voles (*Microtus agrestis*) fed most of the nymphs. Reports of larvae [57, 58] and even a female [57] from lizards and a nymph from a frog [57] are either mistakes, erroneous translations from Russian or accidental infestations [4, 8]. Neumann [59] listed two bat species and also rhinoceros and hippopotamus as hosts. Since these records have never been confirmed by others, they cannot be considered as *bona fide* host-associations.

**Table 1:** Reported hosts of *Dermacentor reticulatus*

Host	Stage^a^	Area	References
Yellow-necked mouse (*Apodemus flavicollis*)	L, N	Europe	[22, 33]
Wood mouse (*A. sylvaticus*)	L, N	Europe	[22, 279, 280]
Striped field mouse (*A. agrarius*)	L, N	Eurasia	[22, 279–281]
Northern birch mouse (*Sicista betulina*)	L, N	Northern Europe, Western Russia	[281]
European pine vole (*Microtus subterraneus*)	L, N	Europe	[22]
Common vole (*Mi. arvalis*)	L, N	Europe, Western Russia	[22, 280, 281]
Narrow-headed vole (*Mi. gregalis*)	L, N	Asia	[279]
Root vole (*Mi. oeconomus*)	L, N	Eurasia	[279, 281]
Field vole (*Mi. agrestis*)	L, N	Europe, Western Russia	[281]
Major’s pine vole (*Mi. majori*)	L, N	Caucasus, North-western Iran	[282]
Bank vole (*Myodes glareolus*)	L, N	Europe, Western Russia	[22, 280, 281]
Northern red-backed vole (*My. rutilus*)	L, N	Eurasia	[279]
European water vole (*Arvicola amphibius*)	L, N	Eurasia	[72]
Eurasian harvest mouse (*Micromys minutus*)	L, N	Eurasia	[281]
European hamster (*Cricetus cricetus*)	L, N	Europe, South-western Russia	[279]
Muskrat (*Ondatra zibethicus*)	L	Eurasia	[279]
Red-cheeked ground squirrel (*Spermophilus erythrogenys*)	L	Asian steppes	[279]
European rabbit (*Oryctolagus cuniculus*)	L, N, A	Europe	[22]
European hare (*Lepus europeus*)	L, N, A	Eurasia	[22, 283]
Common shrew (*Sorex araneus*)	L, N, A	Europe, Russia	[22, 72, 279–281, 284]
Eurasian pygmy shrew (*So. minutus*)	L, N	Europe, Russia	[22, 281]
Eurasian water shrew (*Neomys fodiens*)	L, N	Europe, Russia	[22, 281]
European mole (*Talpa europea*)	L, N	Europe, Western Russia	[281]
Northern white-breasted hedgehog (*Erinaceus roumanicus*)	L, N, A	Eastern Europe, Western Russia	[22, 283]
European hedgehog (*E. europeus*)	N, A	Western Europe	[283]
Least weasel (*Mustela nivalis*)	L, N, A	Eurasia	[22, 279–281]
Stoat (*Mu. erminea*)	N	Eurasia	[279]
European polecat (*Mu. putorius*)	N, A	Europe, Western Russia	[22, 285]
European badger (*Meles meles*)	A	Europe	[286]
Racoon dog (*Nyctereutes procyonoides*)	A	Europe	[140]
Roe deer (*Capreolus capreolus*)	N^b^, A	Europe,	[22, 59, 69, 280, 287, 288]
Fallow deer (*Dama dama*)	A	Europe	[62]
Red deer (*Cervus elaphus*)	N^b^, A	Europe, Western Asia	[22, 59, 279–281, 285, 288, 289]
Moose (*Alces alces*)	A	Eurasia	[68, 289]
European bison or wisent (*Bison bonasus*)	A	Europe, Western Russia	[60, 144, 290–292]
Wild boar (*Sus scrofa*)	A	Eurasia	[22, 59, 61, 70, 285, 289, 293]
Red fox (*Vulpes vulpes*)	A	Eurasia	[22, 31, 61, 286, 294–296]
Golden jackal (*Canis aureus*)	A	Eurasia	[52]
Gray wolf (*Canis lupus*)	A	Eurasia	[42]
Iberian wolf (*Canis lupus signatus*)	A	Iberian Peninsula	[286]
Common starling (*Sturnus vulgaris*)	N^b^	Eurasia	[50]
Blackbird (*Turdus merula)*	N^b^	Eurasia	[50]
Mistle thrush (*Turdus viscivorus*)	L^b^	Eurasia	[50]
Eurasian jay (*Garrulus glandarius*)	N^b^	Eurasia	[22]
Medow pipit (*Anthus pratensis*)	N^b^	Eurasia	[53]
Tree pipit (*Anthus trivialis*)	L^b^,N^b^	Eurasia	[51]
Song thrush (*Turdus philomelos*)	N^b^	Eurasia	[51]
Green sandpiper (*Tringa ochropus*)	N^b^	Eurasia	[51]
Yellow wagtail (*Motacilla flava*)	L^b^	Eurasia	[51]
White wagtail (*Motacilla alba*)	L^b^, N^b^	Eurasia	[51]
Reed bunting (*Emberiza schoeniclus*)	N^b^	Eurasia	[51]
Siberian stonechat (*Saxicola maurus*)	L^b^	Eurasia	[51]
House sparrow (*Passer domesticus*)	L^b^	Eurasia	[51]
Tree sparrow (*Passer montanus*)	L^b^, N^b^	Eurasia	[51]
Pig (*Sus scrofa domesticus* ***)***	A	entire *D. reticulatus* range	[22]
Sheep (*Ovis aries*)	A	entire *D. reticulatus* range	[22, 61, 72, 280, 283, 284, 287]
Goat (*Capra aegagrus hircus*)	L^b^, N^b^, A	entire *D. reticulatus* range	[22, 59, 245, 280, 283, 284, 297]
Cattle (*Bos taurus*)	A	entire *D. reticulatus* range	[60, 61]
Horse (*Equus caballus*)	A	entire *D. reticulatus* range	[60, 61, 69, 245]
Donkey (*Eq. africanus asinus*)	A	entire *D. reticulatus* range	[10]
Cat (*Felis catus*)	A	entire *D. reticulatus* range	[69, 113, 280, 285, 287]
Dog (*Ca. lupus familiaris)*	N^b^, A	entire *D. reticulatus* range	[25, 26, 60, 61, 113, 245, 288, 298]
Human	N^b^, A	entire *D. reticulatus* range	[4, 54–56, 59, 61–66, 69, 280, 284]

^a^L, larva; N, nymph; A, adult

^b^rarely

Adults use an even wider range of host animals (Table 1; Fig. 7). Wild hosts include various cervids, wild boars, foxes, golden jackals, wolves, hedgehogs, hares and rabbits. Domesticated animals are equally important as hosts or even the dominant [25, 60] hosts (e.g. in cities or agricultural areas) for adults and are represented mostly by dogs, horses, donkeys, cattle, buffalo, sheep, goats and pigs [10, 12, 22, 61]. Like immatures, adults possess the adaptive trait to use different vertebrates as dominant (often domesticated) hosts depending on their local availability [60]. Humans can also be occasional hosts of adults [61–66] increasing the public health importance of pathogens harboured by these ticks. The role of immatures in their epidemiology is largely unknown.

Concerning ecological aspects, Nosek [22] has already emphasised that original ecosystems have been changed or greatly affected by human activity across the distribution range of *D. reticulatus*. Although some authors [19] referred to this tick as a species with restricted habitat use*,* on a geographical scale it in fact exists in a wide range of habitat types. These include meadows and open mixed or oak forests [67, 68], clearings [19, 22] river basins, swampy mixed woods, lakeshore vegetation [15, 22, 69], pastured land, heath, scattered scrub, suburban wasteland [31, 70] and coastal dune systems. [5] *Dermacentor reticulatus* is rarely found in closed, dark forests [31] such as the taiga [22, 71] and coniferous forests [72]. It apparently prefers riparian forests (river basins), ecotones between fields and mixed deciduous forests, forest paths and lake shore vegetation [22, 32, 73]. The presence of eyes and the relatively bright and patchy colouration are obvious morphological adaptations to living in open habitats with a relatively high insolation. Its association with wet habitats is clearly shown by its resistance to water. Eggs survive in pools of rainwater [74] and adults remain alive during periodic floods that are often characteristic for their preferred habitats [67]. Accordingly, *D. reticulatus* can also be collected from the common reed (*Phragmites australis*) in wetland habitats [75].

A recent ecological approach [35] found empirical evidence that the niches of *D. reticulatus* and *I. ricinus* segregate along temperature and moisture axes. Based on 25 habitat variables derived from digital maps using GIS for the locations with sympatric populations of the two tick species, *D. reticulatus* appeared to be more thermophilic and hygrophilous than *I. ricinus* while still tolerating large diurnal and seasonal temperature variation. This is not necessarily at variance with the conclusion that *D. reticulatus* is a psychrophilic tick, thriving at relatively low temperatures [76]. Moreover, quantitative evidence suggests that it occurs in places with less precipitation seasonality, near watercourses and water bodies (Široký et al., unpublished data), which further emphasizes its bond to water in the landscape, a feature noted by several observers [22, 42, 76, 77]. Higher tolerance to temperature variation may also explain why it can be encountered along riverbanks and wet grasslands in a cold region of Poland with sunny and hot summers [77] or mountains in Hungary that are often characterised by much higher humidity, especially compared to the lowlands in the Pannonian biogeographical region [34]. The tick’s negative host-seeking activity in response to increasing soil temperatures [76] may thus indicate its higher sensitivity to desiccation relative to *I. ricinus*. Moreover, its larvae are also known to require high relative humidity for successful embryonic development and hatching [78]. Kubelová [35] demonstrated that adult *D. reticulatus* prefers warmer and wetter sites with greater diurnal and seasonal variation in temperature but with less precipitation seasonality than *I. ricinus*. A further difference is that *I. ricinus* seems to be more tolerant of forested habitats than *D. reticulatus*, which prefers open spaces, such as temperate grassland with high moisture conditions, covered by a mosaic of bush and woods [22, 35]. Adults of *D. reticulatus* were shown to survive better in the meadow microclimate than in the forest microhabitat. About 55 % of unengorged females and 58 % of males survived for 399 days in the meadow (including two periods of hibernation), while only 33 % of females and 34 % of males survived in the forest habitat in South Moravia [67].

*Dermacentor reticulatus* has also been observed in urban areas, e.g. in Grenoble, Munich, Warsaw, Lublin, Kiev, Košice and Budapest [31, 50, 75, 79–86]. Although usually absent in downtown parks [87, 88] where larger maintenance hosts are not permanently present, the tick may occur in suburban forests with natural hosts for adults, or even in urbanised areas where dogs (including stray dogs) or horses are common.

### Geographical distribution and recent spread

*Dermacentor reticulatus* is not a newcomer in Europe. A specimen was collected from a fossil woolly rhino (*Coelodonta antiquitatis*) from the Pliocene (extending from 5.33 million to 2.58 million years before present) [89]. It is likely that the distribution patterns of the species have changed over this time and more recently man has likely had a profound effect on the distribution of *D. reticulatus* through the introduction of domestic animals and the alteration of the environment.

**Fig. 8:**
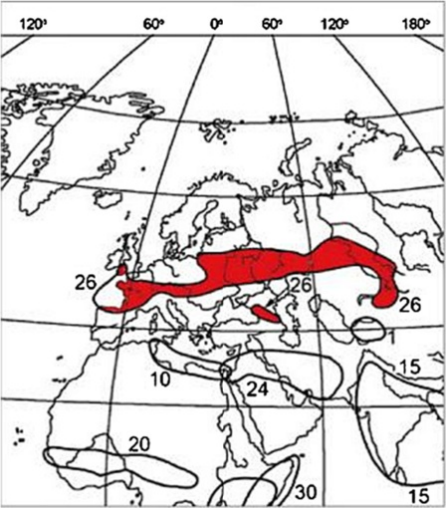
Geographical distribution of *Dermacentor reticulatus* (*red area*, 26) based on Kolonin [92]

*Dermacentor reticulatus* occurs in the western Palaearctic in regions with generally mild climates. Feider [90] published a map showing patchy distribution of the species in Europe from Germany to Bulgaria and in the western part of the former Soviet Union. Immler [19] also included occurrences in western Europe in his map. The world distribution of *D. reticulatus* was completely described for the first time by Kolonin [91]. Based on this publication, its range extends from northern Portugal and Spain in the west to central Asia in the east, forming a quite narrow and long strip in a west-east orientation, with a separate enclave in the Caucasus [91]. The same author published a map (Fig. 8) on the geographical distribution of this species [92]. Although this represents useful information for world-scale studies, the resolution of the map is too low to be applied for regional epidemiological purposes. Currently, *D. reticulatus* receives growing public interest because of its expected increasing epidemiological importance. Therefore, the growing number of studies on its biology, vector competence, and spread helps us to better demarcate its distributional range as a result of the growing number of precise localisations.

**Fig. 9:**
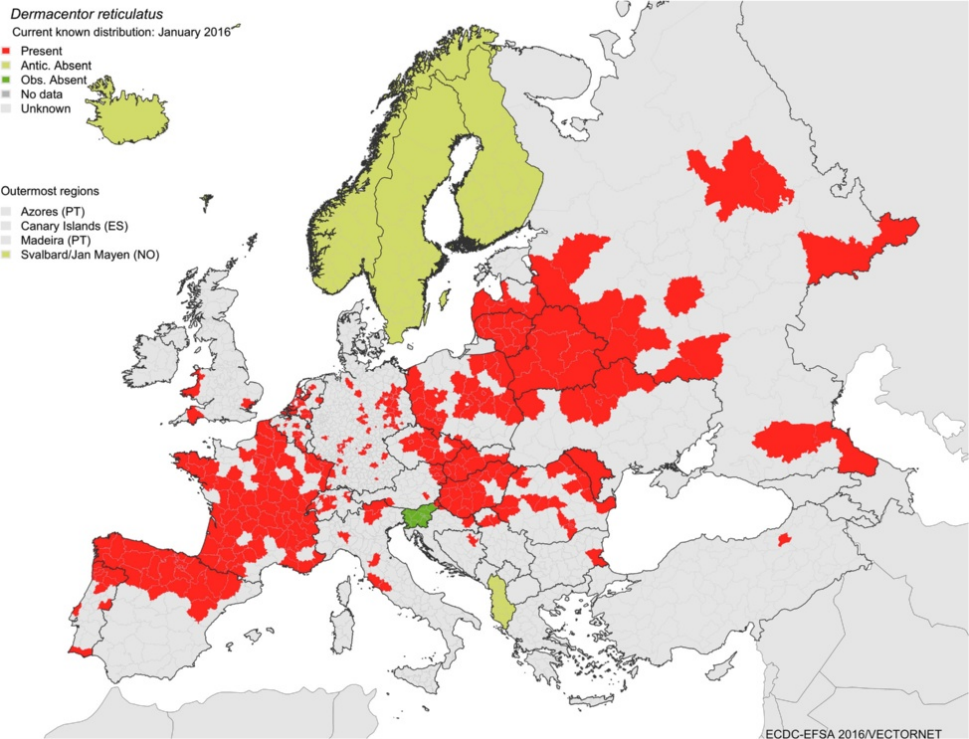
Geographical distribution of *Dermacentor reticulatus* based on the European Centre for Disease Prevention and Control (ECDC) and Vector-Net project. The map shows the current (January 2016) known distribution of the tick species in Europe at ‘regional’ administrative level (NUTS3). They are based on published historical data and confirmed data provided by experts from the respective countries as part of the Vector-Net project; see more at: http://ecdc.europa.eu/en/healthtopics/vectors/vector-maps/Pages/VBORNET-maps-tick-species.aspx#sthash.ca6HyLb6.dpuf

*Dermacentor reticulatus* is absent in the dry Mediterranean climate zone, for example in northern Africa, most of Iberian Peninsula, Italy, the Balkans and Turkey; however, it is present in southern France and Portugal. It is also absent in the cold regions in the north of British Islands, the whole of Scandinavia, and the northern part of the Baltic region. The distribution pattern of *D. reticulatus* seems to be enigmatic even within this frame, being somehow mosaic or highly focal, following ecological requirements of the species. An on-line available map published by the European Centre for Disease Prevention and Control (ECDC) and Vector-Net project (Fig. 9) shows this pattern, however with some impreciseness, for example false occurrence data in the Czech Republic. There are entire districts in the Czech Republic, which are marked on the map; however, there are no published records of *D. reticulatus* occurrence, e.g. from central and eastern Bohemia, Prague, district Vysočina (Pavel Široký, personal communication). Typical foci have to offer proper microclimate with high relative humidity. Open unploughed habitats with high level of ground water in lowlands or low-altitude hills seem to match best its requirements [22, 32]. In higher mountain regions *D. reticulatus* is absent; however, it can occur in climatically favourable valleys.

During the last decades, the distribution of *D. reticulatus* has considerably expanded in some regions. Large areas of north-western and central Europe, formerly thought to be too cold for its survival and completion of its life-cycle, have experienced a remarkable spread of these ticks in Germany, Poland, Hungary, Slovakia, but also the Netherlands and Belgium (for an excellent review see Rubel et al. [93]). The recent climatic changes have been frequently reported as the predominant driving force [62]. However, anthropogenic impact and socioeconomic changes after the fall of the Iron Curtain should not be overlooked [94]. Human activities, agricultural practices in land use, and particularly travelling with animals and animal trade have changed notably during the last decades. For example, increased availability of unploughed open habitats in central Europe with favourable microclimates has enabled settlement of founder engorged female ticks, probably imported on dogs. International motorway stops are also possible hotspots for *D. reticulatus* introduction (Michiel Wijnveld, personal communication) as many people travel by car with dogs. Reforestations and a steady increase in wildlife populations that are appropriate maintenance hosts for the species, may have contributed to the recent spread [82]. The National Game Management Database estimated a two-fold growth of the red fox population and a 5–10 fold growth of populations of wild boar, red, roe, and fallow deer in Hungary during the last five decades [95]. Similar figures have been published for other European countries [62, 69, 82]. A recent study in Poland demonstrated a dynamic expansion of *D. reticulatus* into areas historically free of this species, and underlined the significance of river valleys as important ecological corridors for wildlife [17]. Populations of dogs, one of the most important maintenance hosts, in and around human dwellings are also increasing. According to a 2012 estimation [96], 75.3 million dogs live in European households. The number of stray dogs, that are usually more heavily infested, is estimated to be 100 million in Europe [97]. Increased grazing in natural reserves, together with reduction of pesticide usage might well contribute to the growing population of *D. reticulatus*. From 1965 to 1971, the incidence of tick-borne encephalitis in the former Soviet Union decreased by two- thirds mainly because of the widespread use of DDT (dichloro-diphenyl-trichloroethane) to kill the vector ticks [98]. With the worldwide abandonment of DDT, the incidence of tick-borne encephalitis cases in the former Soviet Union gradually returned to pre-intervention levels within 20 years [99].

**Fig. 10:**
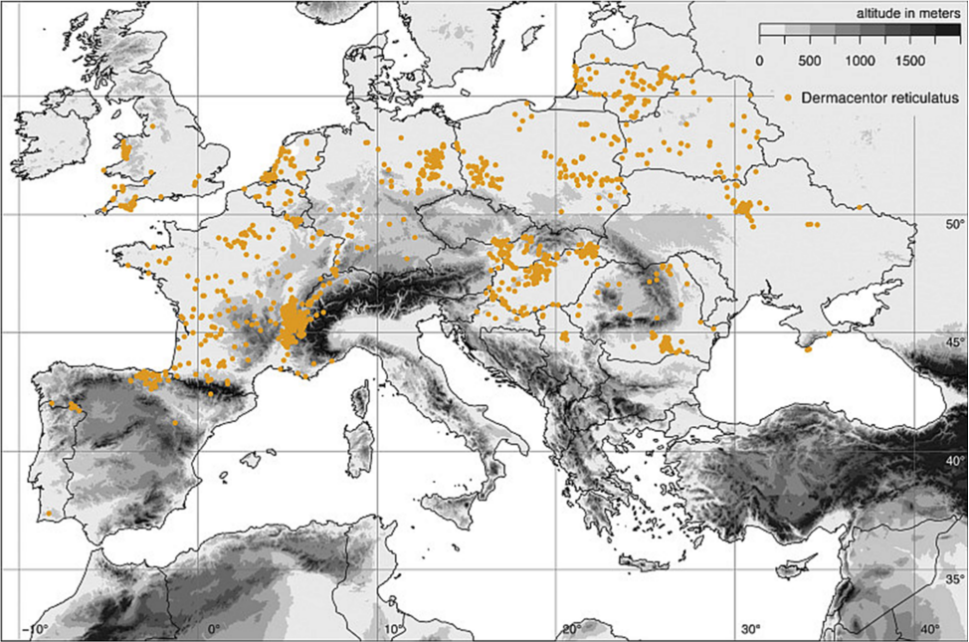
Map of georeferenced *Dermacentor reticulatus* locations based on Rubel et al. [93]

Our knowledge about the tick’s recent distribution depends on the availability of published accurate data. Most of the Iberian Peninsula, the western limit of the range of *D. reticulatus* is covered by unsuitably dry habitats; this explains the absence of this tick in most of Portugal and Spain. Nevertheless, reports from northern administrative regions of Portugal (particularly from Montesinho Natural Park, Braganca district) [100] and from northern Spain (particularly in the Basque Country, Cantabria and Navarre) [101, 102] imply that the tick exists in areas with continental climates [103]. Regarding georeferenced data, France could be considered as a distribution centre in the western Europe [93, 103]. Occurrence is reported throughout this country, including the Pyrenean foothills, the Mediterranean zone, and Biscay areas (Fig. 10) [104–106]. Data on *D. reticulatus* distribution are missing from northern France, particularly along the shore of the English Channel [103]. However, in Belgium and particularly in the Netherlands, the species is reported throughout both countries, including coastal lowlands along the North Sea [69, 107–110]. *Dermacentor reticulatus* is absent from the Alps; however, it penetrates deeply into warmer valleys both in France and western Switzerland, where its occurrence is known for many decades [31, 111–113]. The tick’s north-western limit is located in the United Kingdom. *Dermacentor reticulatus* has been found here for over 100 years and is considered to be endemic, with an apparent recent expansion of its range [114]. Its distribution is restricted particularly to western Wales, Devon and parts of Essex. The tick is apparently absent in Ireland (Fig. 10) [5, 115].

Although central Europe was thought to be free of this species [82] from the Alps in the south, through eastern Switzerland, most of Austria, Slovenia, Czech Republic, Poland, and Germany in the north (nevertheless, without any proof provided by population genetic studies using adequate markers), intensive geographical spread was documented during the last decades in this region. The tick became common within the Pannonian iogeographical region, not only in Hungary [25, 34, 116–118] but also in neighbouring Slovakia [22, 83, 119–121], eastern Austria [122–124] and adjacent areas of the Czech Republic [22, 32, 76, 119, 125]. Focal distribution of *D. reticulatus* has been reported also throughout Germany [62, 126, 127] and recently from Poland [14, 15, 17, 60, 77, 80, 84, 128–131]. Based on this trend, the central European gap in the geographical distribution of the tick may disappear very soon (Fig. 10).

The tick’s range around the Pannonian biogeographical region includes eastern Slovenia [93, 132], northern Croatia [133, 134], and northern Serbia [135, 136]. Ticks have also been occasionally reported from dogs in Bosnia and Herzegovina [133, 137]. In the eastern Balkans, Romania is another example of a rapidly growing number of records of *D. reticulatus* [61, 138, 139]. The tick exhibits an uninterrupted distribution from eastern Poland to Belarus and Baltic countries. Reported distribution is quite even, without remarkable foci throughout Belarus [140, 141], while a bit more clustered in Lithuania and southern Latvia [142, 143]. Local data is absent from the area eastwards of Romania [93], however, we can expect its occurrence in western regions of Ukraine and probably also in Moldavia. Detailed distribution data on *D. reticulatus* have recently been published for areas of central and north-eastern Ukraine, as well as for the Crimean Peninsula (Fig. 10) [50, 85, 93, 144].

The eastern part of *D. reticulatus* distribution was demarcated for the first time by Pomerantsev [71]. The species occurred within the USSR, with its northern limits in the regions of Smolensk, Moscow, Ivanovo, Ryazan, further through Gorki and Kamyshlov area of Sverdlovsk District, Tyumen, Omsk, and Novosibirsk districts, eastwards up to Kansk in Krasnojarsk District. The southern limits extend to the southern Crimean Peninsula, Ciscaucasia and Transcaucasia, eastern Kazakhstan, Kirgizstan, and western Altai Mountains [71]. A similar but more roughly estimated range was depicted in Kolonin [91, 92] (Fig. 8) [91]. Filippova [145] described the eastern part of *D. reticulatus* distribution showing that its occurrence has a disjunctive character, being spread mostly through the southern Taiga in zones of mixed or deciduous forests, from the Baltic region of Kaliningrad, south of Saint Petersburg region, up to the upper reaches of Yenisei River. The species also occurs in the steppe zone along river valleys. Southern limits were established to be in south-western Moldavia, the mountains of the Crimean Peninsula, both Greater and Lesser Caucasus, and northern Kazakhstan. Further, the tick is known from the foothills of Kopet-Dag, Altai, and Tian-Shan Mountains [145]. Recently, the tick and canine babesiosis has been reported from three dogs in the eastern Anatolia region of Turkey [146]. Some areas of the Russian part of the range of *D. reticulatus* have recently been subjected to intensive research resulting in additional distribution data; nevertheless, the exact location, with coordinates, is usually missing [29, 147–151]. China (provinces Xinjiang and even Shaanxi and Shanxi) is considered to be the south-eastern limit of its distribution [152].

### Veterinary health importance

#### Babesia canis

Considering geographical distribution, economic and health impact, *Babesia canis* is undoubtedly the most significant pathogen transmitted to animals by *D. reticulatus.* This piroplasmid apicomplexan parasite is able to invade ovaries of female ticks and is transmitted transovarially to the next generation of larvae [153]. Together with transstadial transmission, this feature enables *D. reticulatus* populations to function as a reservoir in addition to their vector role, enabling maintenance of *B. canis* locally for several tick generations even without a vertebrate reservoir host [154]. A further consequence of the highly specialised *B. canis* life-cycle is that, the key driver of genetic variability of this emerging canine pathogen, the piroplasmid parasite’s exchange of genetic material, occurs within *D. reticulatus* [153].

As reviewed by Matijatko et al [155], the considerable differences in the clinical disease manifestations may also reflect the above mentioned genetic variability leading to different *B. canis* strains. Uncomplicated canine babesiosis (with a mortality rate <5 %) has been suggested to be a consequence of anaemia resulting from haemolysis, whereas complicated canine babesiosis may be a consequence of the development of systemic inflammatory response syndrome (SIRS) and multiple organ dysfunction syndrome (MODS). Clinical signs of uncomplicated babesiosis include pale mucous membranes, fever, anorexia, depression, splenomegaly, hypotension and water hammer pulse. Clinical manifestations of the complicated form of babesiosis (mortality rates of up to 20 %) depend on the particular complications that develop, such as cerebral babesiosis, shock, rhabdomyolysis, acute renal failure, acute respiratory distress syndrome, acute liver dysfunction and acute pancreatitis [155]. A recent study [156] classified *B. canis* strains based on major merozoite surface antigens coding DNA (bc28.1 gene). However, the recognised two groups, Bc28.1-A strains (relatively virulent or mild) and Bc28.1-B (virulent), showed great variation in their geographical distribution. The authors hypothesised that the distribution of *B. canis* genotypes might be dependent on the presence of genetically different *D. reticulatus* strains in certain geographical areas, but this remains to be demonstrated [156]. Such genetic variability and antigenic variation are not only important for the survival of *B. canis* in their vertebrate hosts but has implications for vaccine development strategies. The capacity of *B. canis* to change the antigenic make-up of its merozoite surface is one of the major impediments of vaccine development, and has been suggested as a possible explanation for the limited efficacy of a commercially available vaccine in the field [156, 157].

The wide geographic distribution of *B. canis* is in line with that of its vector, i. e. from western Europe to Siberia [155, 158]. Based on molecular screening of field collected ticks, the prevalence of *B. canis* in adult *D. reticulatus* ticks varies from 0 % (*n* = 197) in studies conducted for instance in Germany [159] or Belarus (*n* = 142) [141] to 0.7 % (*n* = 582) in eastern Poland, 1.64 % (*n* = 855) in the Netherlands [69], 2.3 % (*n* = 1, 205) in south-western Slovakia [120], 3.41 % (*n* = 205) in Ukraine [144], 4.18 % (*n* = 2,585) in Poland [15] to exceptionally as high as 14.7 % (*n* = 327) in eastern Slovakia [120] and 14.8 % (*n* = 233) in southern Poland [15].

The natural cycle of *B. canis* is enigmatic since it has no known wildlife reservoir host. Studies performed on candidate reservoir wild canids did not find evidence for a wild-living host capable of maintaining the parasites. Reports from Italy (205 red foxes, seven grey wolves) [160], Hungary (404 red foxes) [161], Austria (36 red foxes) [162] and Slovakia (nine red foxes) [163] found no *B. canis* despite the large number of wildlife samples screened. Single foxes were found to have *B. canis* infection based on PCR in one of 91 samples in Portugal [164] and one of 73 samples in Bosnia and Herzegovina [165]. This is not surprising as *D. reticulatus* occurs on foxes and can transmit the parasite to this host; however, based on the rarity of infection, the red fox can be excluded as a natural reservoir. Another candidate, the golden jackal (*Canis aureus*) which has spread into new areas recently, has also not been found to be infected in the limited samples tested so far [52, 124, 166]. Captive grey wolves were shown to be susceptible to *B. canis* infection which can be even lethal for them [167], but no evidence exists on their potential role as asymptomatic carriers. There is, therefore, no indication that wolves are capable of playing a role in the natural cycle of this piroplasm. As other *D. reticulatus* hosts were not so far shown to be frequently or at all infected with *B. canis*, the only remaining plausible candidate to fill the gap in the reservoir position of the transmission cycle is the domesticated dog. Our hypothesis is that *B. canis* can persist in some dogs asymptomatically for a long time, so that when infested by *D. reticulatus* serve as a source of the parasite to the feeding ticks. There is empirical evidence for subclinical canine babesiosis, e.g. from France [168, 169], Slovakia [170], Poland [171] and Turkey [172]. However, in order to establish the reservoir role of dogs, experimental infections using xenodiagnostic *D. reticulatus* ticks have to be performed. For the closely related species, *Babesia caballi,* long-term asymptomatic carrier horses have already been reported [173–176].

There are several implications of the probable reservoir role of dogs in the *B. canis* cycle. First, asymptomatic dogs may be able to infect puppies vertically as shown for *Theileria equi* in horses [177]. Although vertical transmission appears to be rare in *Babesia *(*sensu stricto*), it  has been described for *B. divergens* [178]. A recent observation confirmed vertical transmission of *B. canis* from female dogs to puppies [179]. Second, this would provide a sound explanation for the recent geographical spread of canine babesiosis [26, 69, 120, 180]. Based on the relatively low prevalence of the pathogen in field-collected ticks, it is more probable to import a dog with either symptomatic or asymptomatic *B. canis* infection into a new area, than importing infected *D. reticulatus* specimens. When the piroplasm has already been imported with dogs into a new area, the local *D. reticulatus* population is likely to become infected and can sustain *B. canis* for several years by transovarial and transstadial transmission, leading to a detectable presence in the local tick population. Consequently, dogs are not necessarily required for the short term maintenance of infected ticks. In line with this, in many new foci, e.g. in the Netherlands, Belgium, Norway, Switzerland, Hungary, Slovakia, Germany, canine babesiosis was observed first without the presence of infected ticks or even the tick itself in the area [107, 112, 120, 180–185]. Finally, we assume that in evolutionary terms *B. canis* originated in domesticated dogs (or their ancestors) and not in a related wildlife reservoir host.

#### *Babesia caballi* and *Theileria equi*

Equine piroplasmosis caused by *B. caballi* and *T. equi* is the most prevalent tick-borne disease in equids (horses, mules, donkeys, zebras) in certain areas of the world and besides causing important economic losses it also leads to movement restrictions [173]. Worldwide, cases are tracked by the World Organisation for Animal Health (OIE: Office International des Epizooties) (http://www.oie.int/). According to this, most of the equid-inhabited regions of the world are considered endemic for infection and disease. Cases are consistently reported from Central and South America, Cuba, Europe, Asia and Africa. In non-endemic countries such as Australia, Canada, Great Britain, Ireland, Japan, New Zealand, and until recently, the United States, only seronegative horses are allowed to be imported to prevent the introduction of carrier animals [186]. Seropositive horses cannot cross borders to compete in races or horse shows, be used for breeding purposes, or be sold abroad [187]. These two parasites have biological differences but cause similar pathology and have similar vector relationships. Acute disease is characterised by fever, malaise and reduced appetite, increased pulse rates and respiration, anorexia, constipation followed by diarrhoea, tachycardia, petechiae, splenomegaly, thrombocytopenia, and haemolytic anaemia leading to haemoglobinuria and icterus [174, 186]. Horses that recover from acute disease remain persistently infected carriers without overt signs of disease and can be reservoirs for transmission of these protozoan pathogens by vector ticks. Parasitaemia is often too low to be detected on blood smears, but infected animals can be identified by serology or polymerase chain reaction (PCR). Similarly to *B. canis*, sexual-stage development (resulting in genetically new offspring) is completed in ticks for both *T. equi* and *B. caballi* [153, 186].

*Dermacentor reticulatus* is not the only vector species for *B. caballi*, several members of the genera *Hyalomma*, *Rhipicephalus*, *Dermacentor* and *Haemaphysalis* are able to transmit it [174]. The life-cycle of *B. caballi* involves transovarial transmission from females via eggs to hatching larvae. Consequently, *B. caballi* can be sustained for several tick generations similarly to *B. canis*. As *D. reticulatus* is a common ectoparasite of horses [60, 69, 131] and acts as vector of this parasite with transovarial and transstadial transmission [186], it can often infect them with *B. caballi*. This can lead to relatively high seroprevalences of *B. caballi* in endemic areas [173]. Within the world domestic equine population (approximately 112 million in 2013), rates of infection in endemic regions are often above 60 %, and in some regions more than 90 % of the animals are infected with one or both parasites [186]. Most of these are persistently infected without any sign of clinical disease. As suspected for *B. canis*, it has been shown for *B. caballi*, that the basis for its spread is movement of these clinically healthy carrier animals into regions with competent tick vectors, where they can be a source of infection for the naïve horse populations [186]. Once recovered from an acute episode, horses were reported to remain carriers of *B. caballi* for up to four years [174].

*Theileria equi*, previously considered a species of *Babesia*, was reclassified [188] because of the absence of transovarial transmission in the vector and because sporozoites do not infect red blood cells, but first penetrate a lymphocyte (or macrophage) where they develop into schizonts [173]. Infections with *T. equi* (which is more frequently reported [189]), are usually more severe than those with *B. caballi* but it is impossible to distinguish between the two parasitic infections based on clinical signs alone. Equine theileriosis differs from equine babesiosis also in the length of asymptomatic carrier status: once infected, horses remain carriers of *T. equi* for life [174] thereby serving as a continuous source of infection for vector ticks. Similarly to *B. caballi*, *T. equi* can be transmitted by several tick species [186]. The vector competence of *D. reticulatus* for *T. equi,* with experimental evidence of transstadial infection, has been confirmed [190, 191]. The worldwide spread *T. equi* is more prevalent than *B. caballi* [186]; this reflects differences in their vector biology as well as differences in persistence of the parasites in the equine host mentioned above.

#### *Anaplasma marginale*

Bovine anaplasmosis is an important tick-borne disease of domesticated ruminants worldwide caused by infection of cattle with the obligate intraerythrocytic bacterium *Anaplasma marginale* of the family Anaplasmataceae, order Rickettsiales [192]. The acute phase of bovine anaplasmosis is characterised by anaemia, icterus, weight loss, fever, abortion, decreased milk production, and often results in death [193]. Animals surviving the acute phase develop a lifelong persistent infection and can serve as reservoirs for mechanical transmission and biological transmission by ticks [194].

Mechanical transmission occurs in various ways: blood-contaminated fomites, including hypodermic needles, castration instruments, ear tagging devices, tattooing instruments, and dehorning saws or by blood-contaminated mouthparts of biting flies [193–195]. Biological transmission is by ticks and over 20 species have been incriminated as vectors worldwide. Recently, an experimental study has shown that *D. reticulatus* can also transmit *A. marginale* intrastadially [194]. This route of pathogen transmission is enhanced by the extended stay of male *D. reticulatus* ticks on the host and their intermittent feeding behaviour (as detailed above in the section “Life-cycle and ecology”). Males can feed and transmit *A. marginale* multiple times as they transfer among cattle. Indeed *D. reticulatus* can be the main vector of *A. marginale* as shown in a study performed on ticks removed from cattle in Hungary, where *D. reticulatus*, rather than the other three tick species were involved in *A. marginale* transmission [196]. The main route for tick-transmitted bovine anaplasmosis is probably the intrastadial infection by male *D. reticulatus*, since immatures of this tick species usually do not feed on cattle, thus cannot provide transstadial infection for the adult ticks.

### Public health importance

*Dermacentor reticulatus* has been reported parasitising humans in Russia, Austria, the United Kingdom, France, Hungary and Spain [63, 64, 74] but bites humans much less frequently than *I. ricinus* or *I. persulcatus* [63, 197]. It is considered to be the most common [32, 60, 69] or second most common [51, 116] species in many areas and in western Siberia, this species was the second most common tick found on humans after *I. persulcatus* [56]. Based on this, the direct impact of *D. reticulatus* on public health, and its relative contribution to the disease burden caused by vector-borne diseases, is relatively small in many regions of Eurasia, but can be substantial in endemic areas and should definitely not be ignored. An example of emergence as a result of efficient transport by human travel is shown by a recent paper reporting the detection of a male *D. reticulatus* on a patient in Irkutsk (eastern Siberia) who acquired the tick in the Tula region (western Russia), 5,000 km to the west [198]. Even longer journeys have already been made by this species, because its presence was reported on horses transported to the USA in the 1960s, 1970s and 1980s from France [199].

*Dermacentor reticulatus* transmits a particular set of pathogens to humans, which might cause serious disease if not diagnosed and treated appropriately in a timely manner. Awareness by medical doctors of the potential public health risk of this tick in their patient population, and availability of supportive laboratory diagnoses are essential. The pathogens (and associated diseases) that can be transmitted by *D. reticulatus* are briefly reviewed below. The 40 microbial agents that have been detected in this tick are listed, though there is uncertainty about its vector role for some of them (Table 2). It should be noted that molecular techniques have weaknesses, including the inability to distinguish living from dead microorganisms and the risk exists of contamination or PCR artefacts from various sources. Whether *D. reticulatus* can transmit these pathogens should first be established in vector-competence experiments. The unknown relevance of molecular detection of pathogens is exemplified by a study performed on field-collected adult *D. reticulatus* in Poland, where 2.5 % of the 468 ticks were positive for *Babesia microti* [200]. The authors used amplified rDNA fragments that were only 238 base pairs long and the similarity of their PCR products was only 97–99 % to known *B. microti* sequences. Based on these findings, no conclusions should be drawn about the potential vector role or the public health relevance of *D. reticulatus* in transmission of *B. microti*. Czech scientists cultured an additional 38 bacterial strains with mainly unknown medical or veterinary importance from field-collected *D. reticulatus* [201] that are not listed in our table. One of the latest additions to the long list of microbes is *Toxoplasma gondii* [202], a parasite with a life-cycle involving a multitude of hosts but surely not specialised for tick transmission.

**Table 2:** Pathogens detected in *Dermacentor reticulatus*

Status	Pathogen	Disease	Region	Relevance	Note	References
Vector	Omsk haemorrhagic fever virus	Omsk haemorrhagic fever	Western Siberia	PH		[207]
	Tick-borne encephalitis virus	Tick-borne encephalitis	Eurasia	PH		[80, 149, 228, 299]
	*Rickettsia raoultii*	TIBOLA/DEBONEL	Eurasia	PH		[15, 62, 64, 107, 126, 141, 159, 235, 238, 245, 247–249, 300–303]
	*Rickettsia slovaca*	TIBOLA/DEBONEL	Eurasia	PH		[64, 238, 245, 300, 304]
	*Anaplasma marginale*	Bovine anaplasmosis	France	VET	Disseminated infection and mechanical (surgery) calf to calf transmission	[104, 194, 196]
	*Babesia canis*	Canine babesiosis	Eurasia	VET		[69, 112, 123, 144, 180, 300, 305]
	*Babesia caballi*	Equine babesiosis	Southern Europe	VET		[69]
	*Theileria equi*	Equine theileriosis	Eurasia	VET		[188, 306, 307]
Carrier: found in questing or fed ticks or used in experimental infection studies (with unknown vector role)	Kemerovo virus	Kemerovo tick fever	Western Siberia	PH		[148]
	Bluetongue virus (BTV-8)	Bluetongue disease	n.a.	PH	Disseminated infection but no transstadial or transovarial infection	[308]
	Palma virus	?	n.a.	?	transmission by co-feeding on laboratory mice	[309]
	Murid herpesvirus 4	Not known	Slovakia	?		[310]
	*Rickettsia helvetica*	Aneruptive fever, endocarditis	Eurasia	PH		[115, 141, 250]
	*Rickettsia sibirica sibirica*	Siberian tick typhus	Asia	PH		[311, 312]
	*Anaplasma phagocytophilum*	Human, canine and equine granulocytic anaplasmosis	Eurasia	PH + VET		[104, 144, 247, 313, 314]
	*Borrelia burgdorferi* (*s.s*.)	Lyme borreliosis	Eurasia	PH		[141]
	*Borrelia burgdorferi* (*s.l.*)	Lyme borreliosis	Eurasia	PH		[104, 107, 141, 315, 316]
	*Borrelia afzelii*	Lyme borreliosis	Eurasia	PH	Also detected in engorged larvae removed from uninfected mice	[15, 141, 206]
	*Borrelia valaisiana*	Lyme borreliosis	Eurasia	PH		[141]
	*Borrelia garinii*	Lyme borreliosis	Eurasia	PH		[158]
	*Coxiella burneii*	Q-fever	Eurasia	PH		[104, 317]
	*Francisella tularensis* ssp. *holarctica*	Tularemia	Eurasia	PH		[200, 318, 319]
	*Francisella philomiragia*	Opportunistic human pathogen, fish pathogen	Eurasia	PH + VET	Not tick-transmitted	[104]
	*Francisella*-like organisms	Not known	Eurasia	–		[105, 150, 320–324]
	*Bartonella henselae*	Cat scratch disease	Eurasia	PH		[141, 158]
	*Bartonella quintana*	Five-days fever	Eurasia	PH		[158]
	*Bartonella* sp.	?	n.a.	?		[104]
	*Gordonia sputi*	Endocarditis, mediastinitis	Europe	PH	In immunosuppressed individuals, not tick-transmitted	[325, 326]
	*Microbacterium floriorum*	?	Europe	?	In immunosuppressed individuals, not tick-transmitted	[325]
	*Arthrobacter oxydans*	?	Europe	?	Not tick-transmitted	[325]
	*Kocuria kristinae*	Endocarditis, peritonitis	Europe	PH	Not tick-transmitted	[325]
	*Curtobacterium flaccumfaciens*	Septic arthritis	Europe	PH	Not tick-transmitted	[325]
	*Salmonella typhimurium*	Salmonellosis	Eurasia	PH	Not tick-transmitted but transovarially transmitted	[327, 328]
	*Babesia microti*	Human babesiosis	Eurasia	PH		[200, 305]
	*Babesia divergens*	Bovine babesiosis, Redwater fever	Spain	PH + VET		[307, 329]
		Human babesiosis	Europe			
	*Babesia bigemina*	Texas fever	Spain	VET		[307]
	*Theileria* sp. OT1	Not known	Spain	?		[307]
	*Hepatozoon canis*	Canine hepatozoonosis	Worldwide	VET	In engorged nymphs from infected dogs	[298]
	*Toxoplasma gondii*	Toxoplasmosis	Worldwide	PH + VET	Not tick-transmitted	[202]
	*Nosema slovaca*	–	Slovakia, Hungary	–		[330]

*Abbreviations*: *PH* public health; *VET*. veterinary; *n.a.* not applicable

We would like to call attention also to the possible indirect role of this tick species in pathogen cycles. As has been shown for *A. phagocytophilum* and *Babesia microti* transmitted by nidicolous *Ixodes trianguliceps* to rodents [203, 204] and *B. burgdorferi* (*sensu lato*) (*s.l*.) transmitted by *I. hexagonus* to hedgehogs [205], immatures of *D. reticulatus* are probably also involved in the endophilic pathogen cycles of disease agents [206]. Similarly to the recently published endophilic cycle of *B. afzelii* maintained by the nidicolous *I. acuminatus* on rodents and by *I. ricinus* in an exophilic cycle [206], *D. reticulatus* immatures may also maintain pathogens that could be transmitted to humans or domesticated animals by adults of the same species or by other (exophilic) ticks. Exploration of the endophilic pathogen cycles associated with *D. reticulatus* is therefore of fundamental importance.

#### Omsk haemorrhagic fever virus (OHFV)

The first cases of Omsk haemorrhagic fever (OHF) were diagnosed in the 1940s in four adjacent Provinces of Russia: Omsk, Novosibirsk, Kurgan, and Tyumen. Between 1946 and 1958, more than 1,000 cases of OHF were diagnosed, after which the incidence decreased. Although OHF cases have not been officially recorded, an increase of OHF in endemic areas has been apparent since 1988. In about 80 % of cases, OHF infection results in mild flu-like, symptoms. Common symptoms include fever, headache, myalgia, cough, bradycardia, dehydration, hypotension, and gastrointestinal symptoms [207]. Such OHF cases may be easily missed or misdiagnosed [208]. The onset of OHF is sudden, with fever lasting five to 12 days. Approximately 30 to 50 % of patients experience a second febrile phase. During the second phase, patients can develop meningeal signs, but neurological involvement has not been reported. The haemorrhagic manifestations of OHF are typically nosebleeds, bleeding gums, vomiting of blood, blood in the lungs and non-menstrual bleeding of the uterus. Recovery from OHF is generally slow and its case-fatality rate varies from 0.5 to 2.5 % [207].

Omsk haemorrhagic fever virus (OHFV) belongs to the tick-borne virus group, genus *Flavivirus*, family Flaviviridae. It is phylogenetically closely related to tick-borne encephalitis virus (TBEV), and to a lesser extent to Kyasanur forest disease virus (KFDV) and Alkhurma haemorrhagic fever virus (AHFV). It is remarkable that TBEV has spread from western Europe to Japan [209], whereas the circulation of OHFV remained restricted within four Siberian provinces during hundreds of years of evolution [210]. Humans can become infected through tick bites, with *D. reticulatus* being the main vector, or through contact with body fluids of infected animals and environmental samples. The sylvatic cycle of OHFV appears to include several vertebrates, particularly water voles (*Arvicola amphibius*, formerly *A. terrestris*) and narrow-headed voles (*Microtus gregalis*) and the principal vector is *D. reticulatus* which is able to transmit the virus transovarially [7]. Vole populations are cyclic, and expansion of the virus-infected tick population coincides with increases in vole populations [211]. The prevalence of ticks infected with OHF virus corresponds to the density of ticks in a given focus. During the epidemic period (1945-1949) of OHF in the lake region of Omsk district, the density of *D. reticulatus* was ten times greater than during the non-epidemic period of 1959 to 1962. In the former period, all cattle in the region were infested, and larvae and nymphs were mainly found on voles, particularly narrow-headed voles, with the prevalence of infected ticks at 6 %. In contrast, only 0.1-0.9 % of ticks was infected during the non-epidemic period [212].

The disease emerged in Omsk Province shortly after the introduction of the North American muskrat (*Ondatra zibethicus*), when more than 4,000 muskrats were released into the wild. This muskrat species turned out to be highly susceptible to OHFV infection. Many deadly epizootics in muskrats have been recorded since the 1940s. Although OHFV is transmitted mainly by *D. reticulatus*, occupational and recreational activities such as hunting, trapping or skinning muskrats may have also caused OHF outbreaks [208, 213].

Two important unresolved issues remain: (i) What caused the outbreak in the 1940s? Is it really a new pathogen, having sprung up 70 years ago, or an indigenous arbovirus re-emerging as a consequence of new ecological conditions? (ii) What limits the further geographical spread of OHFV? The main vector, *D. reticulatus*, as well as its main vertebrate hosts, the water vole and the muskrat, are widely distributed over northern Eurasia. Perhaps OHFV transmission is only possible in specific climatic conditions, where co-feeding of nymphal and larval stages of *D. reticulatus* occur, which is regarded as a rare event. Another possibility is that other tick-borne flaviviruses, notably TBEV, compete with OHFV. The latter is not unlikely as TBEV protective antibodies cross-react with and neutralize OHFV [214, 215].

#### Tick-borne encephalitis virus (TBEV)

Tick-borne encephalitis (TBE) is a common and occasionally fatal tick-transmitted disease in central and eastern Europe and Russia [216, 217]. It is an infection of the central nervous system caused by the tick-borne encephalitis virus (TBEV). The clinical aspects and epidemiology of TBE, as well the ecological aspects of TBEV have been reviewed elsewhere [218–220], and therefore, they are only mentioned here briefly. The clinical spectrum of the disease ranges from mild meningitis to severe meningoencephalitis with or without paralysis and death. A post-encephalitic syndrome, causing long-lasting morbidity, may occur in patients after acute tick-borne encephalitis. The clinical course and outcome vary by subtype of tick-borne encephalitis virus, age of patients, and host genetic factors [221]. TBEV is transmitted to humans predominantly by *I. ricinus* and *I. persulcatus* and, to a far lesser extent, by *D. reticulatus*. During the last few decades the incidence of the disease has increased and poses a growing health problem in almost all endemic European and Asian countries. Vaccination can effectively prevent the disease and is suggested for persons living in or visiting tick-borne encephalitis endemic areas [222].

Transovarial transmission of the TBEV via the eggs from an infected adult female tick to its offspring has been documented, but seems to be rare and its importance to the maintenance of the virus in nature is considered to be rather low [223]. Compared to, for example, the highly efficient (94–100 %) filial infection rate of *Rickettsia conorii* in *Rhipicephalus sanguineus* [224], the proportion of larvae transovarially infected with TBEV is low (< 5 %) [225]. Although rodents and large mammals (e.g. deer, cattle) can be infected and become viraemic, systemic infection is not necessary and of little importance for viral transmission [226]. TBEV can be transmitted from infected to non-infected ticks when they co-feed in close proximity on the same host [227]. For successful co-feeding transmission nymphs and larvae should feed simultaneously on the same host. It is unlikely that *D. reticulatus* can maintain TBEV in enzootic cycles in Europe as there is only a very short interval for the possibility of co-feeding larvae and nymphs [6] (see also section “Life-cycle and ecology”).

The potential role of *D. reticulatus* in the maintenance and circulation of TBEV and a link with cattle as potential reservoir hosts has been suggested in recent studies from Poland [80, 228]. Mierzejewska et al. [228] recently reported high prevalence of TBEV (7.6 %) in *D. reticulatus* that is consistent with the results (10.8 %) obtained by a previous survey. Interestingly, prevalence of TBEV in *D. reticulatus* may be up to ten times higher than in *I. ricinus* (7–11 % *vs* 0–1.2 %) [228, 229]. Cattle serve frequently as hosts for *D. reticulatus* [10] and the dominance of this tick over *I. ricinus* on bovine hosts in regions endemic for *D. reticulatus* has been reported recently [60]. According to these studies, grazing cattle may play a dual role; they serve as an easily available source of blood meals compared to wild animals, thus supporting the expansion of *D. reticulatus* and might act as a reservoir for the TBEV. Transmission of TBEV to cattle may be followed by transfer of this virus to humans via non- pasteurised milk or other dairy products from infected animals (mainly goats, sheep and cows) [94]. Milk-borne TBE outbreaks or single cases have been reported from Russia, the former Czechoslovakia, Hungary, Austria and Germany [220, 230, 231]. A recent study confirmed that TBEV is transmitted transovarially in *D. reticulatus* [232]. However, the role of *D. reticulatus* compared to that of *I. ricinus* and *I. persulcatus* remains secondary or of local importance in TBEV transmission cycle.

#### *Rickettsia slovaca* and *Rickettsia raoultii*

The two spotted fever rickettsiae transmitted by *D. reticulatus* are *Rickettsia slovaca* and *R. raoultii.* They are the causative agents of the syndromes known as Tick-borne lymphadenopathy (TIBOLA) [233, 234], recently also referred to as Dermacentor-borne-necrosis-erythema-lymphadenopathy (DEBONEL), and Scalp eschar neck lymphadenopathy (SENLAT) [235, 236]. Tick-borne lymphadenopathy is the most common tick-borne rickettsiosis in Europe after Mediterranean spotted fever and occurs in Spain, France, Portugal, Italy, Hungary, Germany, Bulgaria and Poland [235, 236]. Although originally only *D. marginatus* was implicated as a vector, recent studies clarified the role of *D. reticulatus* having a similarly important role in the transmission of rickettsiae causing TIBOLA [64, 236]. The role of male *D. reticulatus* ticks in the transmission of *R. raoultii* has also been shown [64]. In earlier reports, only *R. slovaca* was found to be the main agent of TIBOLA [237, 238], however it seems that *R. raoultii* can be an important or even frequent pathogen in this emerging infection [64].

Clinical manifestations include an eschar at the site of the tick attachment (nearly always on the scalp) surrounded by an erythema and regional/painful lymphadenopathies. If the tick bite is on the scalp, patients may suffer from facial oedema. In rare cases when the tick bite is located elsewhere than the scalp, an erythema with an eschar at the site of the tick-bite usually appears. Reports about these syndromes are rare, but have occurred throughout Europe [235, 239]. Little is known about the enzootic cycles of *R. slovaca* and *R. raoultii*. Probably co-feeding transmission between ticks from the same generation in combination with efficient transovarial transmission may suffice to sustain the enzootic cycle of tick-borne rickettsiae [240]. It is unclear which vertebrate hosts are involved in the amplification of *Rickettsia*-infected ticks, as systemic infection of vertebrate hosts is rarely reported. Contact with horses was found to be an important risk factor for acquiring TIBOLA, however it is not known whether horses might be reservoirs or whether they contribute indirectly with the high number of *Dermacentor* ticks feeding on them [65]. A recent study found *R. slovaca* by PCR in *Apodemus* spp. mice ear biopsies [241]. Another member of the spotted fever group, *R. massiliae* was shown to be transmitted by co-feeding (and possibly mating) between *Rhipicephalus turanicus* ticks [242]. The efficiency of co-feeding transmission of *R. conori* between *Rh. sanguineus* feeding on naïve dogs is estimated to be between 92–100 %, whereas for ticks on seropositive dogs the estimate was between 8–28.5 % transmission via co-feeding [242]. Accordingly, a relatively high prevalence of *R. raoultii* was observed in questing *D. reticulatus* adults in Austria (minimum prevalence of 14.9 %) [243], Romania (18 %) [244], Slovakia (22.3–27 %) [245], UK (27 %) [246], the Baltic countries (1–36.9 %) [247], Germany (44 %) [126], Poland (44–53 %) [15, 248] and Hungary (58 %) [249]. For unknown reasons, *R. slovaca* is found with lower prevalence or not detected at all in this species and occurs more often in *D. marginatus* [245, 250].

#### *Francisella tularensis* and *Coxiella burnetii*

Tularaemia is a zoonosis caused by *Francisella tularensis*, a highly infectious Gram-negative coccobacillus which has been isolated from over 250 species of mammals, birds, reptiles, amphibians, fish and invertebrates. *Francisella tularensis* can be transmitted by several routes, including direct contact with infected body fluids, ingestion of contaminated food or water, inhalation of aerosols and arthropod bites [251]. Ticks have been shown to be infected with *F. tularensis* and *F. tularensis*-like microorganisms and even transstadial transmission has been demonstrated. *Dermacentor reticulatus* has been implicated in the transmission of *F. tularensis* in outbreaks in Russia [252]. However, and most importantly, the incrimination of this vector in transmitting the pathogen to humans has never been proved, and only circumstantial evidence for its vector competence exists [253, 254]. A recent study found no evidence of *F. tularensis* transovarial transmission in *D. reticulatus* [255].

A similar situation holds true for Q fever, which is caused by *Coxiella burnetii.* It can infect a broad spectrum of hosts including livestock, pets, wildlife, birds, fish, reptiles and even invertebrates such as *D. reticulatus* [256, 257], and has several transmission routes. Although other ticks than *D. reticulatus* may readily transmit *C. burnetii* and *F. tularensis* in experimental systems, reports with irrefutable evidence of tick-transmitted Q fever in humans are scarce, if not non-existent [258]. Similarly to the case of *F. tularensis* obscured by *F. tularensis*-like endosymbionts, *Coxiella*-like bacteria are also widespread in ticks and may have been misidentified as *C. burnetii* as emphasised by Duron et al. [258]. Although ticks other than *D. reticulatus* may readily transmit *C. burnetii* in experimental systems, they only occasionally transmit the pathogen in the field. Indirectly, however, *D. reticulatus* may act as entrance for highly infectious agents, such as *F. tularensis* and *C. burnetii,* since bites of this species cause lesions in the host skin [38]. Thus, although transmission of *F. tularensis* and *C. burnetii* by *D. reticulatus* cannot be excluded, other transmission routes to humans play a more important role.

## Conclusions and future challenges

**Fig. 11:**
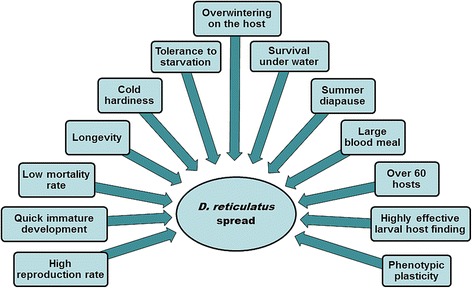
Biological features (intrinsic factors) contributing to successful geographical spread of *Dermacentor reticulatus* (see details and references in the text)

**Fig. 12:**
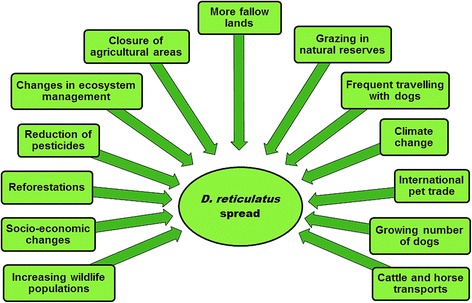
Extrinsic factors contributing to successful geographical spread of *Dermacentor reticulatus* (see details and references in text)

A growing number of reports show that *D. reticulatus* is establishing new foci. We have reviewed the adaptive traits of this species to explain this successful invasion of new areas in the section “Life-cycle and ecology”. The most important biological characters (intrinsic factors) of this species that contribute to the geographical spread are summarised in Fig. 11. Certainly, many driving forces influence the adaptability of a tick species to new areas as reviewed for *I. ricinus* recently [114]. The key extrinsic factors that have enabled the recent spread of *D. reticulatus* were reviewed in the section “Geographical distribution and recent spread” and are summarised in Fig. 12.

Many basic ecological traits of *D. reticulatus* still remain elusive and have to be explored in order to better understand the eco-epidemiological role of this vector species. We do not entirely understand why its larvae are hardly ever found by flagging, while they obviously find their (usual) vole host. Field data to show whether they are mainly inside the rodent nests (endophilous nidicoles) or only near but not within the nests (harbourage nidicoles) [7, 28] do not exist. *Dermacentor reticulatus* larvae in fact cannot be nidicolous by definition, since they can only hatch at places in the leaf litter where the engorged females are randomly dropped off from their hosts and lay eggs, except if gravid females are somehow able to lay eggs in the rodent burrows, which is highly unlikely as also pointed out by Pfäffle et al. [20]. Several alternative and mutually non-exclusive hypotheses can be proposed to explain the host-finding success of larvae despite their lack during sampling via flagging. First, there might be differences in the questing behaviour of *D. reticulatus* larvae compared to *I. ricinus*. Indeed, *D. reticulatus* larvae are able to move faster and they hardly if ever quest on vegetation compared to *I. ricinus* larvae (Hans Dautel, personal communication). Given their relatively high speed and dominantly horizontal movement, they have been shown to occupy a relatively large territory, several square meters [259]. Second, differences in the ecology and behaviour of the dominant host species (voles for *D. reticulatus* and mice for *I. ricinus*) may also account for this observation. A marked ecological difference is that voles build burrows with corridors some centimetres under the surface that often rise into the leaf litter. The nests are 50 cm under the surface in tree trunks, under dead wood or in the ground vegetation [260] and this might further increase the contact rate with larvae preferring leaf litter [20]. In contrast, yellow-necked field mice (*A. flavicollis*) tend to show arboreal occurrence by climbing on trees and nesting in bird nest boxes and tree hollows [261]. As a behavioural difference, yellow-necked field mice move differently through their habitat than bank voles (*My. glareolus*). Short distances are covered by running, while distances longer than two or three metres are covered by jumping, with a jump length up to 80 cm [262]. This can also lead to a dominance of *I. ricinus* (questing higher) and not *D. reticulatus* on mice. A third important factor is that the species structure in small rodent communities in central European floodplain forests is not uniform but changes along the moisture gradient. Bank voles dominate in the lower alluvial plains that hold water for a long time whereas in drier places, mice and voles show the same abundance [263]. Unlike mice, bank voles are capable of acquiring effective resistance against feeding larvae of *Ixodes* spp., resulting in reduced engorged weight and reduced survival of the nymphal stage [264–266]. Finally, field and laboratory experiments may also elucidate a possible host odour preference of larval *D. reticulatus*.

A deeper insight into host associations and their consequences on the eco-epidemiology of pathogens will undoubtedly benefit preventive approaches. The high preference of *D. reticulatus* for dogs together with the appearance of the tick in new areas will surely affect the emergence of certain pathogens too. As in the case of *Dermacentor andersoni* and *Dermacentor variabilis*, the abundant stray dogs and other free ranging dogs largely contributed to the high prevalence of *Rickettsia rickettsii* in ticks and led to hyperendemic foci of Rocky Mountain spotted fever in Mexico and south-west USA [7]. Similarly, the increase of dog populations might easily elevate the risk of infection by some pathogens transmitted by *D. reticulatus*, especially those that are maintained through transovarial transmission by the tick itself [267]. A similar effect exerted by horses was described in the case of TIBOLA infections and contact with horses [65].

Better understanding and mapping of the spread of *D. reticulatus* is pivotal to assess the (local) risk of infections transmitted by this vector species. A joint initiative of the European Food Safety Authority (EFSA) and the European Centre for Disease Prevention and Control (ECDC) resulted in a European network for sharing data on the geographic distribution of arthropod vectors transmitting human and animal disease agents (VectorNet), including *D. reticulatus*. A large database on the presence and distribution of vectors and pathogens in vectors in Europe and the Mediterranean basin is maintained, through a multidisciplinary network of experts and organizations, which (locally) collects these data. An up-to-date map on *D. reticulatus* (Fig. 9) can be found on the ECDC website [268]. Recent maps of this tick’s distribution were published for Poland [17], Germany [127] and Europe [93] (Fig. 10), the digital dataset presented in the latter study are provided on the website http://epidemic-modeling.vetmeduni.ac.at/tickmodel.htm. Enhanced tick surveillance with harmonised approaches for comparison of data enabling follow-up of trends will improve the messages to policy makers, other stakeholders and to the general public on risks related to tick-borne diseases [114].

As reviewed in the veterinary and public health sections, there are a multitude of pathogens that can be transmitted or at least carried by *D. reticulatus*. However, we would like to emphasise that from the pathogen’s point of view, since co-feeding larvae and nymphs are rare, there is a strong selection for pathogens that are transmitted transovarially or that persist in reservoir hosts. For those pathogens that are not transmitted transovarially or cause transient infections in hosts suitable for *D. reticulatus*, this tick species does not play an important role as a vector. As a future research topic, we would like to call attention to the importance of controlled laboratory and field experiments and studies exploring pathogen life-cycles. Molecular biology methods have evolved in an unprecedented way and many laboratories have sophisticated and ever cheaper tools as real-time PCR, microarrays and whole-genome sequencing become available, but fundamental studies clarifying reservoir and vector roles played in pathogen cycles lag behind.

*Dermacentor reticulatus* causes considerable public and veterinary health costs (surveillance, diagnosis and treatment) due to the pathogenic agents transmitted (Table 2). In addition, it can cause losses in livestock production. It has been quantified that the blood amount taken by 100 female *D. reticulatus* causes a loss of at least 400 ml of blood from the host [23]. In heavily infested Belorussian cattle, the average milk yield decreased 2–3 litres per cow [269]. Animal health pharmaceutical companies spend hundreds of millions of Euros on research and development of new products and one of the leading areas is tick control on dogs, very often targeting *D. reticulatus* [270–277]. Dog owners also spend large amounts of money on preventing tick infestations. In the UK alone, the sale figures for ectoparasiticides have doubled in the last decade, exceeding 120 million Euros in 2014 [278]. Despite these financial efforts and the considerable and intensifying research, it seems that due to its adaptive traits, the tick is extending its range and increasing its epidemiologic impact. *Dermacentor reticulatus* is still on the rise.

## Additional files

Additional file 1: Video 1. Male *Dermacentor reticulatus* showing vertical questing behaviour in the laboratory. Field-collected individuals were used on a 30 cm stick. (MP4 3970 kb) Additional file 2: Video 2. Male and female *Dermacentor reticulatus* individuals showing vertical questing behaviour in the laboratory. Field collected individuals were used on a 30 cm stick. (MP4 3679 kb)
